# Thyroid disorders in patients with newly diagnosed rheumatoid arthritis is associated with poor initial treatment response evaluated by disease activity score in 28 joints-C-reactive protein (DAS28-CRP): An observational cohort study

**DOI:** 10.1097/MD.0000000000008357

**Published:** 2017-10-27

**Authors:** Amir Emamifar, Jørgen Hangaard, Inger Marie Jensen Hansen

**Affiliations:** a Department of Rheumatology, Odense University Hospital, Svendborg Hospital, Svendborg; b Faculty of Health Sciences, University of Southern Denmark, Odense; c Department of Endocrinology, Odense University Hospital, Svendborg Hospital, Svendborg; d DANBIO Registry, Copenhagen, Denmark.

**Keywords:** ANA, anti-CCP, DAS28-CRP, IgM RF, rheumatoid arthritis, thyroid disorders

## Abstract

To determine the prevalence of thyroid disorders among newly diagnosed rheumatoid arthritis (RA) patients and evaluate the association between clinical characteristics of RA and thyroid disorders, and also initial treatment response in the RA patients with thyroid disorders.

Newly diagnosed, adult RA patients who were diagnosed according to the new 2010 American College of Rheumatology/European League Against Rheumatism criteria since January 1, 2010, were included. Patients’ demographic data, serology results including immunoglobulin M rheumatoid factor (IgM RF), anticyclic citrullinated peptide antibody (anti-CCP), and antinuclear antibody (ANA), and also disease activity score in 28 joints-C-reactive protein at the time of diagnosis and after 4 months (±1–2 months) of treatment initiation were extracted from Danish Danbio Registry. Patients’ electronic hospital records for the past 10 years were reviewed to reveal if they had been diagnosed with thyroid disorders or they had abnormal thyroid test.

In all, 439 patients were included, female 60.1%, mean age 64.6 ± 15.0 years and disease duration 2.6 ± 1.7 years. Prevalence of thyroid disorders was 69/439 (15.7%) and hypothyroidism was the most frequent disorder (30.4%). The presence of thyroid disorders among RA patients was significantly associated with female sex (*P* < .001), ANA positivity (*P* = .04), and anti-CCP ≥100 EU/mL (*P* = .05). Furthermore, RA patients with thyroid disorders had significantly poorer initial response to RA treatment compared with patients with isolated RA after 4 months of treatment (*P* = .02). There were no associations between thyroid disorders and age, disease duration, and also IgM RF positivity.

Presence of thyroid disorders in RA patients is suggestive of a more aggressive disease and poor outcome, with direct effect on initial treatment response. To diagnose concurrent thyroid disorders at an earlier stage, routine measurement of serum thyroid-stimulating hormone is recommended in all RA patients at the time of diagnosis and with yearly interval thereafter.

## 1 Introduction

Rheumatoid arthritis (RA) is a chronic, autoimmune disease with a prevalence of 0.5% to 1% in the general population. The pathogenesis of RA is still not clearly understood. It may be caused by a complex interaction between genetic and environmental factors resulting in activation of the immune response and synovial inflammation in a distinct symmetric pattern.^[1]^ Even though RA affects mainly joints, extra-articular involvement can occur in approximately 40% of patients.^[2]^

In addition to articular and extra-articular manifestations, various comorbidities can complicate the course of disease.^[3]^ Patients with RA are at increased risk of diabetes,^[4]^ vasculitis,^[5]^ lung diseases^[6]^ and hearing loss,^[7]^ probably due to autoimmunity, compared with the general population. Other common comorbidities among patients with RA are cardiovascular events, different types of cancers, depression, and so on.^[3]^ These comorbid diseases can affect the long-term prognosis and may decline the functional status, and also life span of the patients.^[3]^

An association between RA and thyroid dysfunction with or without autoimmune origin has been reported in 6% to 34% of patients with RA.^[8]^ On the contrary, when presence of thyroid antibodies is considered, despite normal thyroid function, the prevalence can rise up to about 38%.^[9]^ These rates are significantly greater when compared with the general population (about 2–3 times).^[8–11]^ Routine screening of the population for thyroid disease is not recommended; however, assessment of high-risk group, for example, patients with abnormal findings on physical examination, symptoms suggestive of hyperthyroidism or hypothyroidism, women with a positive family history of thyroid disease, previous thyroid dysfunction, and also a history of other autoimmune disease, for example, type 1 diabetes or Addison disease, has been encouraged.^[12]^

Previous studies were in favor of the hypothesis that an association between thyroid dysfunction and RA exists, probably due to autoimmunity; however, the results of these studies were not consistent.^[13]^ Furthermore, most of these studies focused on the clinical characteristic of thyroid dysfunction, and just a few of them discussed the impact of thyroid dysfunction on disease activity and treatment response of RA. The primary objective of this study was to reveal the prevalence of thyroid disorders among newly diagnosed RA patients to find whether thyroid disorders are more prevalent among the RA patients. The secondary objectives of this study were to evaluate the association between clinical characteristic of RA and thyroid disorders, and also to delineate the initial RA treatment response in RA patients with thyroid disorders, because the initial treatment response has an independent prognostic value.^[14–16]^ To the best of our knowledge, this is the first study of its kind investigates the clinical relationship between RA and thyroid disorders.

## 2 Materials and methods

### 2.1 Danbio

The Danish Danbio registry was firstly established in 2000. It provides nationwide data on the disease course of patients with inflammatory rheumatic disease including RA, ankylosing spondylitis, and psoriatic arthritis via unique personal identification code with 2 significant purposes: firstly, helping clinicians to improve quality of routine clinical care by providing a disease chronicle, and secondly, providing a powerful research database. Danbio has been approved by The Danish Data Registry (j. nr. 2007-58-0014 and j.nr. 2007-58-0006) and National Board of Health (j. nr. 7-201-03-12/1), and thereafter, since 2006, it became mandatory to report to the registry. Data are collected from patients and health personnel (nurses and physicians) and are basically divided into baseline variables (eg, demographic data, diagnosis, diseases duration) and longitudinal/follow-up data (eg, treatment, functional status, and disease activity scores).^[17]^ In Denmark, all patients with a newly diagnosis of RA should be registered in Danbio. Each Department of Rheumatology has access to its own patients. At our department, all patients with diagnosis of RA are registered in Danbio at every consultation.

### 2.2 Study design and setting

This is an observational, cohort, single-center study. All parts of the study were performed at the Department of Rheumatology, Svendborg Hospital, Denmark, in December 2015. Ethical approval was obtained from Danish Data Protection Agency (file no.14/50243) and Danish Patient Safety Authority (file no.3-3013-1542/1/).

### 2.3 Participants

All adult patients diagnosed with RA, registered in Danbio by rheumatologists at our department during outpatient visits, were considered to enter into the study. The diagnosis of RA was established according to the new 2010 American College of Rheumatology (ACR)/European League Against Rheumatism (EULAR) criteria for RA.^[18]^ Inclusion criteria were as follows: patients who were registered at the Department of Rheumatology, Svendborg hospital, Denmark; adult patients who were diagnosed with RA since January 1, 2010, according to the new criteria. Patients who passed away or were referred to the other departments were also included. Patients who were diagnosed with RA before January 1, 2010, and juvenile RA were excluded from the study.

### 2.4 Initial rheumatoid arthritis treatment

The initial treatment for all newly diagnosed RA patients at our department is methotrexate, with increasing doses up to 25 mg per week, depending on disease activity score in 28 joints-C-reactive protein (DAS28-CRP) as an index of disease activity. Furthermore, treatment can be supplemented by hydroxychloroquine and sulfasalazine, and also prednisolone (given intramuscular, intra-articular, or orally) in case of remaining inflammation. The treatment goal is to achieve remission, that is, DAS28-CRP less than 2.6 (or low disease activity, ie, DAS28-CRP ≤3.2) as quickly as possible.

### 2.5 Data collection

Patients’ demographic data (age, sex, year of diagnosis), serology test results including immunoglobulin M rheumatoid factor (IgM RF), anticyclic citrullinated peptide antibody (anti-CCP), and antinuclear antibody (ANA), and also DAS28-CRP at the time of diagnosis and after 4 months (±1–2 months) of treatment initiation were collected. The electronic hospital records of the patients for the past 10 years were evaluated, to the extent data were available, for a positive history of thyroid disorders and also use of thyroid medications and abnormal thyroid laboratory tests (triiodothyronine [T3], thyroxine [T4], thyroid-stimulating hormone [TSH]) to clarify if the patients have been diagnosed with thyroid disorders (hypothyroidism, hyperthyroidism, goiter, thyroiditis, autoimmune disease) as well. Thyroid peroxidase antibody (TPOAb), thyroglobulin antibody (TGAb), and thyrotropin receptor antibody (TRAb) were used to delineate thyroid autoimmunity. The individual medical records from family physicians were not examined in this study, because we did not have access to such records. Diagnoses of thyroid disorders subgroups were made at the Department of Endocrinology based on routine follow-up and updated guidelines. Patients with subclinical thyroid disease, who had increased or decreased TSH, but normal T3 and/or T4 and no clinical symptoms, were also included in the statistical analysis.

### 2.6 Variables

Demographic data were extracted from Danbio. The results of IgM RF (normal range: <15 IU/mL), anti-CCP (normal range: <20 EU/mL), and ANA (normal range: <1.0 IU) were collected and analyzed both quantitative and qualitative (positive/negative). DAS28-CRP and ΔDAS28-CRP were calculated as follows:

DAS28-CRP = 0.56^∗^√(Tender Joint) + 0.28^∗^√(Swollen Joint) + 0.36 × ln(CRP+1) + 0.014 × Global Visual Analog Scale + 0.96.

ΔDAS28-CRP = DAS28_1_-CRP (at the time of diagnosis) − DAS28_4_-CRP (after 4 months of treatment initiation ± 1–2 months). The lower reporting limit of CRP was considered as <10 mg/L.^[19]^

In our laboratory, the following reference values were used: TSH (0.3–4 mIU/L), T3 (1.3–2.2 nmol/L), T4 (60–130 nmol/L), TPOAb (2–10 × 10^3^ IU/L), TGAb (<60 × 10^3^ IU/L), and TRAb (<0.7 IU/L).

### 2.7 The EULAR response criteria

According to the EULAR response criteria an improvement of DAS28-CRP greater than 1.2 in a patient with a present score of ≤3.2 was defined as good response. Patients with an improvement of >0.6 to ≤1.2 and a present score of ≤5.1, or an improvement of >1.2 and a present score of greater than 3.2, was considered to respond moderately to treatment. Nonresponder patients are categorized as those who had an improvement of ≤0.6 regardless of DAS28-CRP score, or an improvement of >0.6 to ≤1.2 and a present score of >5.1.^[20]^ Results of ΔDAS28-CRP were also assessed by the EULAR response criteria.

### 2.8 Statistical analysis

All statistical analyses were performed using Microsoft Excel 2010. Continuous data were presented as mean ± standard deviation (±SD), and categorical data as frequencies and respective percentages. Comparisons of the above mentioned variables, between RA patients with and without thyroid disorders, were made with Student *t* test. We used 1-way analysis of variance (ANOVA) test to elucidate any differences of mean ΔDAS28-CRP in subgroups of thyroid disorders. When comparing 2 binary variables, the chi-square test was performed. *P* value was considered as significant if *P* ≤ 0.05. In case of missing data, we used pairwise deletion to keep as many cases as possible for each analysis.

## 3 Results

Of 974 RA patients registered in the regional part of Danbio, 439 patients had a diagnosis of RA based on the new 2010 ACR/EULAR criteria and fulfilled the inclusion criteria. Five hundred thirty-five patients, who were diagnosed with RA before January 1, 2010, were excluded from the study. Demographic and disease characteristics of included patients are summarized in Table 1. The prevalence of thyroid disorders was 69/439 (15.7%).

**Table 1:**
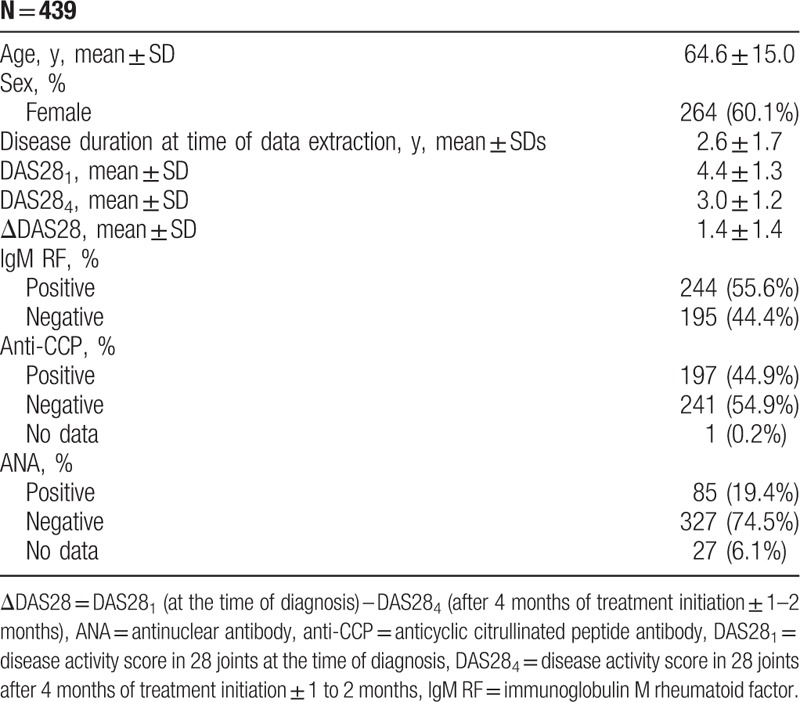
Demographic and disease characteristics of the included patients.

The standard treatment was methotrexate, eventually combined with hydroxychloroquine and sulfasalazine, if needed. In cases of severe inflammation, prednisolone (intra-articular/intramuscular/oral) was added to the treatment regimen.

The results of our study demonstrated that presence of thyroid disorders among RA patients is significantly associated with female sex, ANA positivity, and anti-CCP ≥100 EU/mL. Furthermore, RA patients with thyroid disorders had significantly poorer initial response to the RA treatment compared with patients with isolated RA. There was no association between thyroid disorders and age, disease duration, and also IgM RF positivity (Table 2).

**Table 2:**
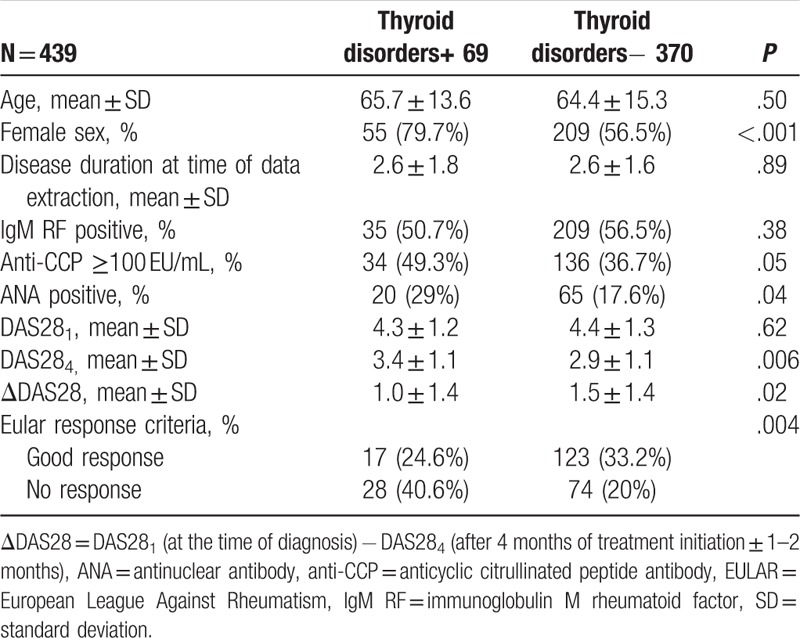
Association of thyroid disorders with sex, disease duration, IgM RF, anti-CCP, ANA, ΔDAS28, and EULAR response criteria in rheumatoid arthritis patients in each group.

Hypothyroidism was the most common thyroid disorder with a prevalence of 21/69 (30.4%). There was no significant difference of mean ΔDAS28-CRP in subgroups of thyroid disorders (Table 3).

**Table 3:**
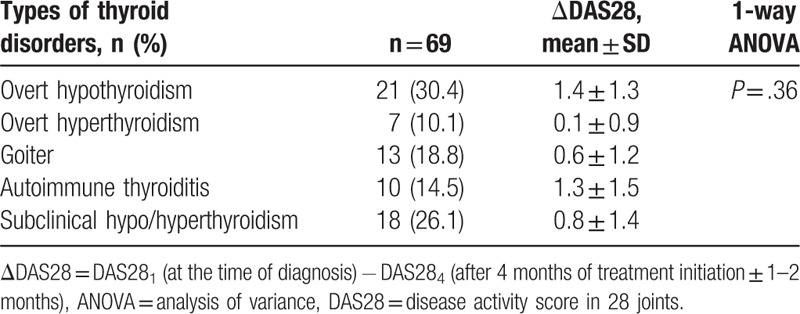
Subgroups of thyroid disorders in the rheumatoid arthritis patients together with ΔDAS28 for each subgroup.

## 4 Discussion

This was the first cohort study, of its kind, evaluating the initial response to treatment among RA patient with and without thyroid disorders. The key results of our study were as follows: the prevalence of thyroid disorders among our RA patients who had been in contact with the hospital in the central part of Denmark was 15.7% that was higher compared to the general Danish population^[21,22]^; the presence of thyroid disorders in RA patients was significantly associated with female sex, ANA positivity, and anti-CCP ≥100 EU/mL; and the initial treatment response was significantly poorer among RA patients with thyroid disorders, regardless of thyroid disorders subgroups, compared with the patients with isolated RA that was of great interest in this study.

Our results were consistent with previous findings confirming that thyroid disorders were more prevalent in patients diagnosed with RA^[8]^; however, there were some inconsistencies. A study by Bianchi et al^[23]^ showed that thyroid disorders presented irrespective of disease activity. This probably occurred due to different disease activity assessment. Shiroky et al in a cohort study of 119 RA patients, evaluating the association of thyroid dysfunction in RA, did not find any association between thyroid disorders and age, RF, and also ANA.^[10]^ Our results also confirmed that there was no association between presence of thyroid disorders in RA patients and age, and also IgM RF; however, we think that small difference between our results and Shiroky et al's results was caused by methodological or laboratory analysis differences. Our results were in line with a recent study by Joshi et al^[24]^ and illustrated that there was a significant correlation between disease activity (at the time of diagnosis) and thyroid disorders among RA patients. A population-based, case-control study (1998 adult cases vs 2252 controls) by Bengtsson et al^[25]^ revealed that patients treated with thyroxin (hypothyroidism) were at double risk of both anti-CCP negative (odds ratio [OR] 2.1, 95% confidence interval [CI] 1.5–3.1) and anti-CCP positive (OR 1.9, 95% CI 1.4–2.6) RA. Cárdenas Roldán et al^[9]^ found the prevalence of autoimmune thyroid disease equal to 9.8% in a cross-sectional study which evaluated 800 RA patients for concurrent autoimmune thyroid disease.

In a study by Koszarnyet al,^[26]^ evaluating the relationship between antithyroid antibody titers and selected parameters of RA activity in 75 consecutive hospitalized patients with RA, significant positive correlations was observed between TPOAb and DAS28, and also TGAb, and both CRP and erythrocyte sedimentation rate (ESR). Apart from this, DAS28 was significantly higher among RA patients with TPOAb/TGAb positive compared with patients with TPOAb/TGAb negative.

The relationship between female sex and thyroid disorders among RA patients in this study was in agreement with the more frequent female sex in both RA and thyroid disorders. The significant association between presence of thyroid disorders in RA patients and ANA positivity, and also anti-CCP ≥100 EU/mL was suggestive of a more aggressive disease with poor outcome in these patients. Earlier studies also revealed the expressive correlation between aggressive RA and high anti-CCP titer, and also ANA positivity.^[27–29]^ Furthermore, statistically significant difference between DAS28-CRP after 4 months of treatment initiation and ΔDAS28-CRP among RA patients with and without thyroid disorders supported this point of view.

The effect of RA treatment on the thyroid autoimmunity is little known. However, benefits of biological treatment, that is, tumor necrosis factor (TNF) inhibitors to regulate and even depress thyroid autoimmunity, has been discussed recently, which delineates the possible role of TNF-α in a shared pathology between RA and thyroid autoimmunity. A study by Raterman et al, on 138 consecutive adalimumab-treated patients with RA who were naive for TNF-blocking agents, resulted in decreasing thyroid autoantibodies, that is, TPOAb and also TSH. The authors concluded that TNF inhibitors could improve thyroid function in hypothyroid patients with RA (especially in patients who were L-thyroxine-naive and TPOAb+).^[30]^ Furthermore, TNF inhibitors may regulate expression of proinflammatory cytokines and apoptosis in thyroids, leading to reduce level of inflammation, earlier resolution, and decreased fibrosis.^[31]^ Further investigations are still needed to enlighten the effect of TNF inhibitors on thyroid autoimmunity.

It has been accepted that early diagnosis of RA along with initiating treatment at early stage of the disease is decisive to prevent further joint destruction. The first few months after treatment initiation are critical for long-term outcome.^[14]^ The lower disease activity achieved at 6 months will result in the better long-term outcome. A clinical remission within 3 to 6 months, regardless of treatment regime, stops the progression of joint damage.^[15,16]^ In fact, the concept of 4 months follow-up and ΔDAS28-CRP in the current study was implemented on the significance of first few months after treatment initiation. However, longer follow-up period, for example, at 6 or 12 months, is worth considering in future studies.

The shared susceptibility genes involved in the pathogenesis of RA and thyroid disorders are HLA gene complex including HLA-DR-B1 (human leukocyte antigen DR B1), CTLA 4 (cytotoxic T-lymphocyte-associated antigen 4), PTPN22 (protein tyrosine phosphatase non-receptor type 22), FCRL3 (Fc receptor-like 3), and IL2RA (interleukin 2 receptor subunit alpha).^[32]^ On the contrary, previous studies elucidated that the carriership of HLA gene complex is associated with anti-CCP positivity and therefore more aggressive disease. In this study, the higher percentage of anti-CCP ≥100 EU/mL in RA patients with thyroid disorders not only supports the role of genetic factors in the pathogenesis of thyroid disorders and RA, but also explains the poorer initial response to the RA treatment in these patients.^[33]^

This study had some limitations as we only selected newly diagnosed RA patients (based on the new 2010 ACR/EULAR criteria) between January 2010 and December 2015, selection bias might be occurred. This is especially relevant for the association of thyroid disorders in RA patients and disease duration, since 1 of the possible hypothesis could be that patients with longer duration of disease are exposed to higher rate of comorbidities, for example, thyroid disorders compared with newly diagnosed patients. The above mentioned bias can be prevented by including all RA patients regardless of diagnosis year. On the contrary, the individual medical records form family physicians were not examined in this study since patients with mild disease may not being referred to the hospital. In addition, thyroid disorders diagnosed longer than 10 years ago were not identified in this study. Therefore, the prevalence of thyroid disorders might have been even higher. The results of this study have a high degree of generalizability due to a feasible sample size and broad inclusion criteria.

Our results indicate that the presence of thyroid disorders can indirectly affect the prognosis of RA, possibly because of the poorer treatment response. We propose routine measurement of serum TSH in all RA patients at the time of diagnosis and with yearly interval thereafter. This strategy will detect thyroid disorders at an earlier stage, leading to early treatment initiation and possibly better prognosis.

## Acknowledgment

We thank Mrs Maryam Mousavi for her contribution to data collection.
